# Supplementary material for: Whole-genome sequencing analysis identifies rare, large-effect noncoding variants and regulatory regions associated with circulating protein levels
Source: Nat Genet. 2025 Feb 24;57(3):626–34. doi: 10.1038/s41588-025-02095-4 (PMC11906349; doi:10.1038/s41588-025-02095-4)
Supplement: Supplementary file 1 — Supplementary Information [file 41588_2025_2095_MOESM1_ESM.pdf]

nature genetics

Supplementary information https://doi.org/10.1038/s41588-025-02095-4

Whole-genome sequencing analysis identifies rare, large-effect noncoding variants and regulatory regions associated with circulating protein levels

In the format provided by the authors and unedited

Supplementary Information

Analysis of Coding Variants

The average effect of predicted loss-of-function variants was -1.80 SD, equating to a reduction of raw circulating protein levels to approximately half (47.3%), with some notable exceptions (**Supplementary Table 7**). The estimated effects of predicted loss-of-function variants were weaker towards the 3' ends of the gene, consistent with variants in the last exon escaping nonsense mediated decay, (**Extended Data Fig. 2**). Missense variants were associated with a weaker effect, reducing circulating protein levels by 15.7% (-1.05SD) on average (**Extended Data Fig. 2),** and the average effect of synonymous variants was closer to zero (-0.463SD).

We identified 8 rare variants which, despite being annotated as loss-of-function, were associated with increased circulating protein levels (**Supplementary Table 7**). The eighty-two carriers of the splice region variant 8:23028330:C:T were unusually consistently affected, such that they generate a secondary, bimodal peak in raw TNFRSF10B measurement levels. On the raw scale, the splice variant was associated with more than 140x the mean protein levels (beta = 144.1 [143.8, 144.4], $P < 1 \times 10^{-300}$), which was consistent across all carriers. The 1bp deletion was not associated with such dramatic changes on the raw scale (beta = 2.67 [2.15, 3.20], $P = 3.31 \times 10^{-23}$).
